# The Association Between Urinary Tract Infection and Overactive Bladder Treatment

**DOI:** 10.3389/fphar.2021.803970

**Published:** 2022-01-25

**Authors:** Kuang-Ming Liao, Ka-Lok Lio, Yu-Ju Chou, Chen-Chun Kuo, Chung-Yu Chen

**Affiliations:** ^1^ Department of Internal Medicine, Chi Mei Medical Center, Chiali, Taiwan; ^2^ Department of Pharmacy, Centro Hospitalar Conde de São Januário, Macau Health Bureau, Macau SAR, China; ^3^ Department of Pharmacy, Chiayi Chang Gung Memorial Hospital, Chiayi, Taiwan; ^4^ Master Program in Clinical Pharmacy, School of Pharmacy, Kaohsiung Medical University, Kaohsiung, Taiwan; ^5^ Department of Pharmacy, Kaohsiung Medical University Hospital, Kaohsiung, Taiwan; ^6^ Department of Medical Research, Kaohsiung Medical University Hospital, Kaohsiung, Taiwan

**Keywords:** overactive bladder, pharmacoepidemiology, anti-muscarinic agents, mirabegron, persistence, adherence

## Abstract

**Background:** Overactive bladder (OAB) syndrome is defined as urinary urgency, with or without urge incontinence in the absence of an underlying pathological or metabolic cause. Treatment for OAB involves anti-muscarinic agents and beta 3-adrenoceptor agonists. As a previous study showed that treatment may increase the risk of urinary tract infection (UTI), we conducted a nationwide, population-based, retrospective study to assess UTI risk associated with OAB medication adherence, and different types of OAB medication.

**Methods:** The source of data was medical records from National Health Insurance Research Database (NHIRD). Patients who were diagnosed with OAB in outpatient records from January 1, 2014 to December 31, 2016 were included. Outpatient visits included an attendance at primary care or the emergency department. The index date was the first prescription medication for OAB treatment after diagnosis. The targeted population was those diagnosed with OAB, and targeted drugs were anti-muscarinic agent (including flavoxate, oxybutynin, propiverine, solifenacin, tolterodine, and trospium) and mirabegron. Adherence was assessed based on the proportion of days covered in 12 months among mirabegron and anti-muscarinic agents. A multivariate Cox proportional-hazards model was used to compare the risk of UTI with OAB medication adherence, and different types of OAB medication.

**Results:** There were 39,975 outpatients diagnosed with OAB in the database from 2014 to 2016. Excluding those younger than 20 years old and for whom the information was incomplete in the database, 21,869 patients were included in the final OAB cohort. Overall, risk of UTI was not influenced by the targeted drugs or adherence during the follow-up period, regardless of UTI history or sex.

**Conclusion:** OAB is a common problem in Taiwan. After 12 months of follow-up, there was no difference between anticholinergic medications and beta-3 agonists, nor between high and low adherence in the risk of UTI.

## Introduction

Overactive bladder (OAB) syndrome is defined by the International Continence Society (ICS) as urgency, with or without urge incontinence, usually with increased daytime frequency and nocturia in the absence of pathologic or metabolic factors that would explain these symptoms (Nik-Ahd et al., 2018). OAB also is high in prevalence and commonly affects patients over the age of 40. The prevalence of those with OAB aged 40–44 years and older than 60 years is 10.8 and 27.9%, respectively, in Asia (Chuang et al., 2019). Despite the fact that OAB is a nonfatal disease, the symptoms may not be curable. In general, the symptoms impact the patient’s quality of life and are more likely to induce other diseases, such as urinary tract infection (UTI) (Nik-Ahd et al., 2018) or fracture (Szabo et al., 2018).

According to guidance from the American Urological Association/Society of Urodynamics, Female Pelvic Medicine and Urogenital Reconstruction, there are four lines of treatment to manage OAB (Lightner et al., 2019). The first-line treatment for OAB is behavioral treatment. Second-line treatments are medication and include anti-muscarinic agents and beta 3-adrenergic receptor agonist.

Anti-muscarinic agents approved for OAB and covered by the Taiwan National Health Insurance (NHI) program include oxybutynin, flavoxate, tolterodine, solifenacin, trospium, and propiverine (Athanasopoulos and Giannitsas, 2011). Mirabegron is a beta-3 adrenergic receptor agonist that causes relaxation of the detrusor smooth muscle during the storage phase of the urinary bladder and increases the bladder’s storage capacity, thereby reducing feelings of frequency and urgency (Takasu et al., 2007). To assess the long-term safety and efficacy of mirabegron and solifenacin in combination compared with monotherapy, a multicenter, randomized, double-blind, phase 3 trial (SYNERGY II) of patients with OAB symptoms including frequency and urgency with incontinence for more than 3 months reported treatment-emergent adverse events, such as UTI (Gratzke et al., 2018).

The mechanism of medication and UTI is not clear. There is documented data on mirabegron as well as solifenacin for a decrease of the volume of urinary retention, but some physicians may consider UTI risk when prescribing mirabegron and solifenacin. As uncontrolled symptoms of OAB may also induce infection, it is important to balance the treatment benefit when considering the risk of UTI with medication. The aim of our study was to examine the hazard of UTI with OAB medication adherence and comparing risk of UTI between anti-muscarinic agents and beta 3-adrenoceptor agonists.

## Materials and Methods

### Data Source

Taiwan launched a single-payer National Health Insurance Program in 1995. The insurance program currently covers up to 99.7% of Taiwan’s population (Hsing and Ioannidis, 2015). In 2002, the government of Taiwan created a database, the National Health Insurance Research Database (NHIRD), to collect claim data from the NHI for research purposes.

Claim records of the full population database from January 1, 2014 to December 31, 2016 were accessed. The available data included outpatient, inpatient, and emergency visit records, prescription records, beneficiaries’ information, and death records.

This study was approved by the IRB of Kaohsiung Medical University Chung-Ho Memorial Hospital (KMUHIRB-EXEMPT(I)-20180041). Because patients’ data could not be identified, the requirement for informed consent was waived.

### Study Design and Population

We conducted a nationwide, population-based retrospective study to estimate the incidence and hazard of outpatient visits or hospitalization due to UTI in the OAB population with different OAB medication adherence or different OAB medication exposure in Taiwan. The cohort included OAB patients stratified by whether the patients were diagnosed with UTI and using antibiotics within 1 year before the index date. Adherence to targeted drugs was divided into high and low adherence in 12 months. The patients were followed up for 1 year to assess UTI events.

### Identification of the Overactive Bladder Cohort

#### Inclusion Criteria

Patients who were diagnosed with OAB (International Classification of Diseases, Ninth Revision [ICD-9] codes 59651 and ICD, 10th Revision [ICD-10] codes N3281) in outpatient records from January 1, 2014 to December 31, 2016 were included. The index date was the first prescription for medication to treat OAB after diagnosis. Considering the coverage by NHIs in Taiwan, we defined anti-muscarinic agents (including flavoxate, oxybutynin, propiverine, tolterodine, solifenacin, and trospium) and beta 3-adrenoceptor agonists (mirabegron) as targeted drugs. For patients who accepted the targeted drug in the observation period, the first drug prescribed was defined as the index drug.

### Exclusion Criteria

1) Age less than 20 years old.
2) Data incomplete in Registry for Beneficiaries.
3) Diagnosed with stress incontinence or mixed incontinence within 365 days before the index date.
4) Accepted a procedure or surgery for relieving OAB symptoms within 1 year before the index date.
5) Died within 1 year after the index date.
6) Accepted any targeted drugs within 1 year before the index date.
7) Used combination therapy at the index date.
8) Did not accept any targeted drug.
9) UTI diagnosed within 30 days before follow-up (to avoid interference from previous infection).

#### High and Low Adherence

Adherence was measured by the proportion of days covered (PDC) within 12 months after the index date. According to a previous study, PDC≥0.8 represents the high-adherence group, whereas PDC<0.8 represents the low-adherence group (Karve et al., 2009).

#### Urinary Tract Infection History

We classified the population based on two indicators: UTI history and sex.

Patients diagnosed with UTI within 30 days before the start of the follow-up date were excluded to avoid interference from the last infection.

The remaining patients were assigned to a group depending on the diagnosis and medication records from 1 year before the index date to the start of the follow-up date. Patients diagnosed with UTI and prescribed systematic antibiotics regardless of inpatient or outpatient visit were classified as the ever had a UTI history group. In contrast, patients who did not have a UTI diagnosis record were classified as never having UTI history group. This stratification was performed because patients with a disease history were more likely to experience related infections again (Foxman, 2014).

#### Sex

Sex was the second stratification variable because the risk of UTI differs physiologically between males and females. Therefore, we stratified by sex to analyze the incidence rate and risk. According to UTI history and sex, the cohort for analyses focused on four different groups: ever had a UTI history group and never had a UTI group in males and females.

### Outcomes

#### Definition of Event

The main events were outpatient visits or hospitalization due to UTI. We defined the start follow-up date as 1 year after the index date and the follow-up period as 1 year. In sensitivity analysis, we included no medication for comparison.

#### Measurement of Covariates

The main covariates included baseline characteristics, comorbidities, and comedication. Baseline characteristics included age, sex, and insurance premium. Age and sex were collected at the index date.

#### Statistical Analyses

In descriptive statistics, categorical variables were analyzed by chi-square tests, and the results are presented as percentages. Continuous variables, which are presented as the mean (SD), were analyzed by Student’s *t*-test. Incidence was estimated as the total number of events divided by the total person-years. The risk of UTI was calculated by Cox regression with time-dependent variables. The multivariate model was adjusted for baseline characteristics, comorbidities, and comedication. To reduce the influence of interaction and collinearity between covariates, a stepwise model was used to assess important factors. The probability for stepwise entry was 0.001, and that for removal was 0.05. The entry probability was calculated by dividing the number of adjusted factors by 0.05 to solve the collinearity problem. All the above analyses were performed with SAS 9.4 software. Statistical significance was two-tailed and *α* = 0.05.

#### Sensitivity Analyses

In subgroup analysis, we modified the cutoff point of high adherence as PDC ≧0.5 and low adherence as PDC<0.5 (Hsing and Ioannidis, 2015). The targeted drugs can be classified into two mechanisms: anti-muscarinic agents and beta 3-adrenoceptor agonist drugs. Our study compared the risk of UTI between these two mechanisms of drugs.

## Results

There were 39,975 outpatients diagnosed with OAB in the database from 2014 to 2016. After excluding those aged younger than 20 years old and for whom information was incomplete in the database, 38,538 patients were included in the OAB cohort. As outlined in the exclusion criteria, 2488 patients were diagnosed with mixed incontinence or stress incontinence, and 108 had ever accepted intradetrusor injection onabotulinum toxin A, operation, and incontinence stimulation; 626 patients died during the 1 year after the index date. Overall, 1794 patients accepted targeted drugs for OAB, and 358 were using combination therapy on the index date.

After screening medical records within 30 days before the start of follow-up, 602 patients were diagnosed with UTIs. The remaining 21,869 patients were evaluated for risk of UTI. We stratified the population by UTI history and sex, with 6212 (28.41%) and 15,687 (71.59%) patients in the UTI history and never had a UTI history groups, respectively. There were 905 (14.57%) males and 5307 (85.43%) females in the UTI group (Table 1) and 7530 (48.00%) males and 8127 (51.81%) females in the never had a UTI group (Table 2).

**TABLE 1:** Baseline characteristics of the cohort, as stratified by sex and adherence in the ever had UTI history group.

Characteristics	Male cohort			Female cohort		
	Low adherence *N* = 842	High adherence *N* = 63	*p* value	Low adherence *N* = 5174	High adherence *N* = 133	*p* value
Demographic						
Age, mean (SD)	59.98 (19.26)	69.76 (15.27)	<0.0001	55.78 (16.41)	67.38 (16.28)	<0.0001
Age group (%)	—	—	0.0046	—	—	<0.0001
20 ≤ age<40	167 (19.83)	5 (7.94)		967 (18.69)	8 (6.02)	
40 ≤ age<60	193 (22.92)	8 (12.70)		2003 (38.71)	32 (24.06)	
60 ≤ age<80	328 (38.95)	31 (49.21)		1792 (34.63)	57 (42.86)	
80 ≤ age	154 (18.29)	19 (30.16)		412 (7.96)	36 (27.07)	
Comorbidities (%)						
Hypertension	372 (44.18)	39 (61.90)	0.0095	1738 (33.59)	74 (55.64)	<0.0001
Dyslipidemia	225 (26.72)	20 (31.75)	0.4723	1393 (26.92)	43 (32.33)	<0.0001
Diabetes mellitus	202 (23.99)	18 (28.57)	0.5058	944 (18.25)	39 (29.32)	<0.0001
IBS	87 (10.33)	2 (3.17)	0.1050	460 (8.89)	10 (7.52)	0.4549
Drug abuser	14 (1.66)	2 (3.17)	0.3076	24 (0.46)	0 (0.00)	1.0000
Obesity	4 (0.48)	0 (0.00)	1.0000	19 (0.37)	1 (0.75)	0.2762
Chronic kidney disease	58 (6.89)	6 (9.52)	0.4404	150 (2.90)	11 (8.27)	<0.0001
Chronic liver disease	105 (12.47)	3 (4.76)	0.1055	643 (12.43)	18 (13.53)	0.0255
Insomnia	196 (23.28)	15 (23.81)	1.0000	1430 (27.64)	36 (27.07)	0.0058
Anxiety	160 (19.00)	11 (17.46)	0.8928	1315 (25.42)	26 (19.55)	0.3384
Depression	58 (6.89)	5 (7.94)	0.7955	562 (10.86)	17 (12.78)	0.0131
Dementia	43 (5.11)	6 (9.52)	0.1400	189 (3.65)	19 (14.29)	<0.0001
Bipolar disorder	14 (1.66)	0 (0.00)	0.6155	59 (1.14)	0 (0.00)	1.0000
Schizophrenia	24 (2.85)	0 (0.00)	0.4032	49 (0.95)	2 (1.50)	0.1964
Alzheimer’s disease	2 (0.24)	1 (1.59)	0.1948	12 (0.23)	6 (4.51)	<0.0001
BPH	482 (57.24)	51 (80.95)	0.0004	—	—	—
Ureteral obstructions	212 (25.18)	13 (20.63)	0.5133	689 (13.32)	21 (15.79)	0.0043
Hematuria	120 (14.25)	8 (12.70)	0.8777	710 (13.72)	18 (13.53)	0.0708
Other bladder disorder	75 (8.91)	5 (7.94)	0.9747	429 (8.29)	21 (15.79)	<0.0001
Neuromuscular disorder	84 (9.98)	7 (11.11)	0.9428	532 (10.28)	26 (19.55)	<0.0001
Chronic cystitis	19 (2.26)	2 (3.17)	0.6525	214 (4.14)	9 (6.77)	0.0094
Urological tumor	61 (7.24)	8 (12.70)	0.1346	84 (1.62)	7 (5.26)	0.0006
Urethral disorder	29 (3.44)	2 (3.17)	1.0000	134 (2.59)	3 (2.26)	0.4839
Acute cystitis	69 (8.19)	3 (4.76)	0.4655	1282 (24.78)	28 (21.05)	0.1175
Urethral infection	45 (5.34)	3 (4.76)	1.0000	338 (6.53)	5 (3.76)	1.0000
Pyelonephritis	19 (2.26)	3 (4.76)	0.1929	203 (3.92)	10 (7.52)	<0.0001
Comedication (%)						
Alpha blocker	269 (31.95)	39 (61.90)	<0.0001	232 (4.48)	15 (11.28)	<0.0001
5 alpha reductase inhibitors	26 (3.09)	5 (7.94)	0.0578	—	—	—
CCB	88 (10.45)	23 (36.51)	<0.0001	332 (6.42)	48 (36.09)	<0.0001
Imipramine	38 (4.51)	5 (7.94)	0.2144	216 (4.17)	15 (11.28)	<0.0001
Duloxetine	2 (0.24)	1 (1.59)	0.1948	21 (0.41)	1 (0.75)	0.2992
Dicyclomine	5 (0.59)	1 (1.59)	0.3522	34 (0.66)	2 (1.50)	0.1128
Hyoscyamine	2 (0.24)	0 (0.00)	1.0000	14 (0.27)	2 (1.50)	0.0264

IBS, irritable bowel syndrome; BPH, benign prostatic hyperplasia; UTI, urinary tract infection; CCB, calcium channel blocker.

**TABLE 2:** Baseline characteristics of the cohort, as stratified by sex and adherence in the never had a UTI history group.

Characteristics	Male cohort			Female cohort		
	Low adherence *N* = 6799	High adherence *N* = 731	*p* value	Low adherence *N* = 7891	High adherence *N* = 236	*p* value
Demographic						
Age, mean (SD)	58.15 (17.39)	70.34 (11.88)	<0.0001	57.53 (15.98)	69.77 (14.63)	<0.0001
Age group (%)	—	—	<0.0001	—	—	<0.0001
20**≤**age<40	1213 (17.84)	13 (1.78)		1201 (15.22)	7 (2.97)	—
40**≤**age<60	2047 (30.11)	115 (15.73)		2990 (37.89)	55 (23.31)	—
60**≤**age<80	2816 (41.42)	441 (60.33)		3005 (38.08)	103 (43.64)	—
80**≤**age	723 (10.63)	162 (22.16)		695 (8.81)	71 (30.08)	—
Comorbidities (%)						
Hypertension	2755 (40.52)	430 (58.82)	<0.0001	2819 (35.72)	142 (60.17)	<0.0001
Dyslipidemia	1917 (28.20)	253 (34.61)	0.0003	2281 (28.91)	78 (33.05)	0.1904
Diabetes mellitus	1398 (20.56)	210 (28.73)	<0.0001	1444 (18.30)	64 (27.12)	0.0008
IBS	635 (9.34)	51 (6.98)	0.0412	772 (9.78)	15 (6.36)	0.1005
Drug abuser	85 (1.25)	8 (1.09)	0.8523	31 (0.39)	0 (0.00)	1.0000
Obesity	32 (0.47)	5 (0.68)	0.4007	38 (0.48)	0 (0.00)	0.6283
Chronic kidney disease	312 (4.59)	44 (6.02)	0.1011	212 (2.69)	11 (4.66)	0.1037
Chronic liver disease	909 (13.37)	92 (12.59)	0.5919	874 (11.08)	25 (10.59)	0.8984
Insomnia	1302 (19.15)	184 (25.17)	0.0001	2031(25.74)	68 (28.81)	0.3231
Anxiety	1043 (15.34)	119 (16.28)	0.5395	1781 (22.57)	45 (19.07)	0.2336
Depression	430 (6.32)	56 (7.66)	0.1875	727 (9.21)	26 (11.02)	0.4077
Dementia	225 (3.31)	48 (6.57)	<0.0001	248 (3.14)	29 (12.29)	<0.0001
Bipolar disorder	65 (0.96)	3 (0.41)	0.2019	77 (0.98)	3 (1.27)	0.5053
Schizophrenia	93 (1.37)	9 (1.23)	0.8674	105 (1.33)	4 (1.69)	0.5601
Alzheimer’s disease	15 (0.22)	3 (0.41)	0.4102	28 (0.35)	4 (1.69)	0.0132
BPH	3302 (48.57)	576 (78.80)	<0.0001	**—**	**—**	**—**
Ureteral obstructions	769 (11.31)	71 (9.71)	0.2142	554 (7.02)	18 (7.63)	0.8183
Hematuria	342 (5.03)	34 (4.65)	0.7206	461 (5.84)	13 (5.51)	0.9406
Other bladder disorder	427 (6.28)	43 (5.88)	0.7322	459 (5.82)	23 (9.75)	0.0174
Neuromuscular disorder	285 (4.19)	37 (5.06)	0.3133	652 (8.26)	45 (19.07)	<0.0001
Chronic cystitis	57 (0.84)	1 (0.14)	0.0659	160 (2.03)	6 (2.54)	0.4858
Urological tumor	289 (4.25)	80 (10.94)	<0.0001	101 (1.28)	10 (4.24)	0.0014
Urethral disorder	56 (0.82)	4 (0.55)	0.5619	96 (1.22)	5 (2.12)	0.2199
Acute cystitis	96 (1.41)	7 (0.96)	0.4023	1141 (14.46)	19 (8.05)	0.0074
Urethral infection	66 (0.97)	1 (0.14)	0.0381	277 (3.51)	3 (1.27)	0.0935
Pyelonephritis	14 (0.21)	1 (0.14)	1.0000	55 (0.70)	1 (0.42)	1.0000
Comedication (%)						
Alpha blocker	2211 (32.52)	492 (67.31)	<0.0001	279 (3.54)	21 (8.90)	<0.0001
5 alpha reductase inhibitor	273 (4.02)	89 (12.18)	<0.0001	**—**	**—**	**—**
CCB	735 (10.81)	246 (33.65)	<0.0001	594 (7.53)	77 (32.63)	<0.0001
Imipramine	292 (4.29)	79 (10.81)	<0.0001	317 (4.02)	33 (13.98)	<0.0001
Duloxetine	15 (0.22)	5 (0.68)	0.0385	26 (0.33)	4 (1.69)	0.0105
Dicyclomine	56 (0.82)	9 (1.23)	0.3568	63 (0.80)	4 (1.69)	0.1297
Hyoscyamine	12 (0.18)	1 (0.14)	1.0000	15 (0.19)	2 (0.85)	0.0858

IBS, irritable bowel syndrome; BPH, benign prostatic hyperplasia; UT, urinary tract infection; CCB, calcium channel blocker.

Regarding the UTI history group, high-adherence patients were significantly older than low-adherence patients, regardless of sex. In males, hypertension and benign prostate hypertrophy were more prevalent in high-adherence patients. In females, irritable bowel syndrome, obesity, anxiety, bipolar, schizophrenia, hematuria, urethral disorder, and acute cystitis were not significantly different between the high- and low-adherence groups. With respect to comedication, the high-adherence group was significantly more likely to be prescribed alpha blockers and calcium channel blockers, for both sexes in Table 1.

In the group of never had a UTI history, high-adherence patients were significantly older than low-adherence patients, regardless of sex. Regarding comorbidities, the prevalence of hypertension, diabetes, dementia, and urological tumors was greater in the high-adherence group, in both sexes. We also examined comedication, and the high-adherence group was more likely to be prescribed alpha blockers, 5 alpha reductase inhibitors, calcium channel blockers, imipramine, and duloxetine. The baseline characteristics of the cohort stratified by sex and adherence are given in Table 2.

### Risk of Outpatient Visits Due to Urinary Tract Infection

In the UTI history group, 129 (15.32%) low-adherence and 6 (9.52%) high-adherence male patients experienced UTIs and sought care in the outpatient setting during the 1-year observation period. The rate was 24.51 per 100 person-years in the low-adherence group and 13.99 in the high-adherence group. In the female cohort, there were 1279 (24.72%) low-adherence and 30 (22.56%) high-adherence patients, with a mean follow-up time of 0.610 and 0.630 person-years, respectively. The rate was 40.51 per 100 person-years in the low-adherence group and 35.83 per 100 person-years in the high-adherence group. Overall, high-adherence patients had a lower incidence rate of outpatient visits due to UTIs than low-adherence patients, regardless of sex. Cox regression analysis estimated the risk of outpatient visits, which was 0.517 (*p* value = 0.1343) and 0.833 (*p* value = 0.3352) times in males and females, respectively, after adjusting for covariates in Table 1.

In males with never had a UTI history, 222 (3.27%) low-adherence and 24 (3.28%) high-adherence patients experienced UTIs with outpatient visits, for rates of 4.47 per 100 person-years and 4.65 in the low- and high-adherence groups, respectively. Among females, 785 (9.95%) low-adherence and 22 (9.32%) high-adherence patients were found, with incidence rates of 14.44 and 13.84 per 100 person-years, respectively. After adjusting for covariates in Cox regression, the risk was 0.723 (*p* value = 0.1463) and 0.970 (*p* value = 0.8901) times in males and females, respectively. Risk of mild UTI in the four stratified groups did not differ significantly between the high- and the low-adherence groups in Table 2.

#### Risk of Hospitalization Due to Urinary Tract Infection

For the UTI history group, in males, 45 (5.34%) low-adherence and 2 (3.17%) high-adherence patients had UTI upon admission, with rates of 7.91 and 13.17 per 100 person-years, respectively. In the female cohort, there were 129 (2.49%) low-adherence patients and 8 (6.02%) high-adherence patients, and the mean follow-up time was 0.728 and 0.701 person-years, respectively. After calculation, the rate was 33.97 per 100 person-years in the low-adherence group and 32.18 in the high-adherence group. Based on Cox regression analysis, the estimated risk of outpatient visits after adjusting for covariates was 0.467 (*p* value = 0.3361) and 1.228 (*p* value = 0.6003) times in males and females, respectively in Table 1.

For the never had a UTI group, 83 (1.22%) low- and 10 (1.37%) high-adherence male patients had UTI upon admission, and the rates were 1.65 and 1.92 per 100 person-years, respectively. In addition, we found 121 (1.53%) low-adherence and 11 (4.66%) high-adherence female patients; the incidence rates were 2.11 and 6.67 per 100 person-years, respectively. The risk was 0.647 (*p* value = 0.2090) and 1.706 (*p* value = 0.1126) in males and females, respectively, in Cox regression adjusted for covariates. The risks of severe UTI in the four stratified groups were not significantly different between the high-adherence group and the low-adherence group, though the risk was higher in females. The detailed information is shown in Table 2.

#### Sensitivity Analyses: Adherence

We modified the cutoff point of adherence as 0.5 in the PDC indicator, with no change in the trend. Except for the never had a UTI group of females, the high-adherence group had a 1.819 times higher risk of admission due to UTIs after adjusting for covariates (*p* = 0.0236) (Table 3 and Table 4).

**TABLE 3:** Risk of UTI (outpatient) in sensitivity analysis of the cutoff point of adherence.

Cutoff point of adherence = 0.5	cHR	95% CI	*p* value	aHR^a^	95% CI	*p* value	sHR	95% CI	*p* value
Risk of outpatient visits due to UTI									
Ever had a UTI history group									
Male									
Low adherence	1.000	(Reference)	—	1.000	(Reference)	—	1.000	(Reference)	—
High adherence	0.832	(0.507–1.367)	0.4886	0.718	(0.419–1.231)	0.2284	0.708	(0.395–1.268)	0.2451
Female									
Low adherence	1.000	(Reference)	—	1.000	(Reference)	—	1.000	(Reference)	—
High adherence	1.185	(0.946–1.483)	0.1396	1.123	(0.889–1.418)	0.3318	1.130	(0.891–1.434)	0.3124
Never had a UTI group									
Male									
Low adherence	1.000	(Reference)	—	1.000	(Reference)	—	1.000	(Reference)	—
High adherence	1.308	(0.965–1.772)	0.0835	0.970	(0.700–1.343)	0.8540	0.969	(0.698–1.344)	0.8494
Female									
Low adherence	1.000	(Reference)	—	1.000	(Reference)	—	1.000	(Reference)	—
High adherence	0.856	(0.634–1.157)	0.3120	0.846	(0.622–1.150)	0.6220	0.839	(0.618–1.140)	0.2613

UTI, urinary tract infection.

**TABLE 4:** Risk of UTI (hospitalization) in sensitivity analysis of the cutoff point of adherence.

Cutoff point of adherence = 0.5	cHR	95% CI	*p* value	aHR^a^	95% CI	*p* value	sHR	95% CI	*p* value
Risk of hospitalization due to UTI									
Ever had a UTI history group									
Male									
Low adherence	1.000	(Reference)	—	1.000	(Reference)	—	1.000	(Reference)	—
High adherence	1.521	(0.756–3.058)	0.2397	1.526	(0.688–3.388)	0.2987	1.532	(0.640–3.669)	0.3384
Female									
Low adherence	1.000	(Reference)	—	1.000	(Reference)	—	1.000	(Reference)	—
High adherence	3.241	(2.036–5.159)	<0.0001	1.819	(1.084–3.052)	0.0236	1.831	(1.067–3.144)	0.0282
Never had a UTI group									
Male									
Low adherence	1.000	(Reference)	—	1.000	(Reference)	—	1.000	(Reference)	—
High adherence	1.214	(0.734–2.010)	0.4503	0.677	(0.395–1.162)	0.1573	0.678	(0.384–1.198)	0.1813
Female									
Low adherence	1.000	(Reference)	—	1.000	(Reference)	—	1.000	(Reference)	—
High adherence	2.461	(1.514–4.0001)	0.0003	1.579	(0.949–2.627)	0.0786	1.582	(0.983–2.546)	0.0589

a Adjusted for demographic variables, comorbidities, and comedication.

PY, person-year; cHR, crude hazard ratio; aHR, adjusted hazard ratio; sHR, subdistribution hazard ratio; CI, confidence interval; UTI, urinary tract infection.

#### Comparison of Risk Between Targeted Drugs With Different Mechanisms

The risk between mirabegron and anti-muscarinic agents was not significantly different, regardless of outpatient visits and hospitalization. The detailed information is shown in Table 5 and Table 6.

**TABLE 5:** Risk of UTI (outpatient) in sensitivity analysis of drugs with different mechanisms.

	cHR	95% CI	*p* value	aHR^a^	95% CI	*p* value	sHR	95% CI	*p* value
Risk of outpatient visits due to UTI									
Ever had a UTI history group									
Male									
Mirabegron	1.000	(Reference)	—	1.000	(Reference)	—	1.000	(Reference)	—
Antimuscarinic agents	1.548	(0.723–3.316)	0.2605	1.876	(0.841–4.185)	0.1245	1.925	(0.853–4.343)	0.1146
Female									
Mirabegron	1.000	(Reference)	—	1.000	(Reference)	—	1.000	(Reference)	—
Antimuscarinic agents	1.214	(0.922–1.599)	0.1666	1.225	(0.926–1.621)	0.1558	1.223	(0.930–1.634)	0.1456
Never had a UTI group									
Male									
Mirabegron	1.000	(Reference)	—	1.000	(Reference)	—	1.000	(Reference)	—
Antimuscarinic agents	1.041	(0.635–1.705)	0.8741	1.326	(0.804–2.187)	0.2689	1.336	(0.817–2.185)	0.2485
Female									
Mirabegron	1.000	(Reference)	—	1.000	(Reference)	—	1.000	(Reference)	—
Antimuscarinic agents	1.293	(0.861–1.942)	0.2155	1.260	(0.835–1.902)	0.2717	1.266	(0.837–1.914)	0.2636

cHR, crude hazard ratio; aHR, adjusted hazard ratio; sHR, subdistribution hazard ratio; CI, confidence interval; UTI, urinary tract infection.

**TABLE 6:** Risk of UTI (hospitalization) in sensitivity analysis of drugs with different mechanisms.

	cHR	95% CI	*p* value	aHR^a^	95% CI	*p* value	sHR	95% CI	*p* value
Risk of hospitalization due to UTI									
Ever had a UTI history group									
Male									
Mirabegron	1.000	(Reference)	—	1.000	(Reference)	—	1.000	(Reference)	—
Antimuscarinic agents	1.915	(0.464–7.900)	0.1108	3.836	(0.754–19.515)	0.1020	3.939	(0.530–29.252)	0.1902
Female									
Mirabegron	1.000	(Reference)	—	1.000	(Reference)	—	1.000	(Reference)	—
Antimuscarinic agents	1.373	(0.562–3.353)	0.4868	1.398	(0.558–3.502)	0.4743	1.409	(0.549–3.618)	0.4759
Never UTI group									
Male									
Mirabegron	1.000	(Reference)	—	1.000	(Reference)	—	1.000	(Reference)	—
Antimuscarinic agents	0.996	(0.461–2.154)	0.9920	1.395	(0.637–3.055)	0.4054	1.397	(0.628–3.109)	0.4128
Female									
Mirabegron	1.000	(Reference)	—	1.000	(Reference)	—	1.000	(Reference)	—
Antimuscarinic agents	1.842	(0.586–5.791)	0.2961	1.914	(0.601–6.100)	0.2720	1.925	(0.613–6.051)	0.2623

a Adjusted for demographic variables, comorbidities, and comedication.

PY, person-year; cHR, crude hazard ratio; aHR, adjusted hazard ratio; sHR, subdistribution hazard ratio; CI, confidence interval; UTI, urinary tract infection.

## Discussion

Our study evaluated UTI hazard in the OAB population associated with OAB medication adherence, and different types of OAB medication in Taiwan. The study result showed that high OAB medication adherence did not increase the risk of UTI than low adherence according to outpatient visits or hospitalizations. Furthermore, anticholinergic medications and beta-3 agonists had similar hazard of UTI after 1 year of follow-up. After sensitivity analysis of modifying the cutoff point of adherence of 0.5, the risk of outpatient visits still did not differ significantly among the four stratifications. Nevertheless, females in the UTI history group had a 1.819-fold higher risk of admission than those in the low-adherence group (*p* = 0.0236).

OAB and UTI have similar symptoms of urgency and incontinence (Abrams et al., 2002). UTI is common among women, and diagnosis of OAB in women should exclude UTI, diabetes mellitus, and neurogenic voiding dysfunction. Bacterial infection damages the urinary tract epithelium, and the number of suburothelial and urothelium inflammatory cells increases. These inflammatory cells initiate an inflammatory cascade response and release inflammatory mediators, resulting in hypersensitivity to bladder distension (Ke et al., 2021). Kuo et al. (Kuo et al., 2010) showed that acute bacterial cystitis increases urinary nerve growth factor levels, and that antibiotic treatment improves OAB symptoms and significantly decreases urinary nerve growth factor levels in patients with symptomatic cystitis. Overall, urinary nerve growth factor levels were significantly diminished in OAB patients who received antimuscarinic treatment compared with those given placebo. Bladder biopsies were performed in women with recurrent UTI, with lower E-cadherin, stronger mast cell expression, higher numbers of apoptotic cells, and increased expression of tryptase and Bax in recurrent UTI compared with normal control specimens (Chuang and Kuo, 2013). These results demonstrated that chronic inflammation and damage to the barrier function of urothelial cells due to apoptosis lead to recurrent UTI in women.

Human detrusor relaxation is controlled by the cyclic adenosine monophosphate pathway, which can be triggered via activation of *β*-ARs by noradrenalin. In the human bladder, *β*3-Ars account for more than 95% of all *β*-AR mRNAs and are the major *β*-ARs mediating human detrusor relaxation (Andersson and Arner, 2004). Mirabegron acts on OAB via detrusor relaxation in the storage or filling phase of the micturition cycle and reduces neurogenic detrusor activity. Because of its mechanism of action, mirabegron has also been reported to increase the risk of UTI in post-marketing reports of urinary retention (Sacco and Bientinesi, 2012). The mechanism of action of mirabegron in UTI is not well proven and previous studies (Andersson and Arner, 2004; Sacco and Bientinesi, 2012) were not based on functional urological data but only on pharmacological data on isolated muscle preparations. Also, the clinical trial studies (Athanasopoulos and Perimenis, 2005; Athanasopoulos et al., 2008; Athanasopoulos, 2010; Chapple, 2010; Füllhase et al., 2010) data do not give good insights since data on UTI in similar patient groups do not find different percentages of UTI in patients treated with antimuscarinics and patients on mirabegron. Only in patients on combination treatments were more UTI observed which suggests that the combination jointly lowers the threshold for sensitivity to UTI.

Antimuscarinic agents are important medications for treating OAB. However, there is a risk of urinary retention that is particularly important in patients with prostate hyperplasia. Previous studies have examined urine retention and increased residual volume in patients with OAB who received antimuscarinic agent treatment, but no strong relationship was detected (Athanasopoulos and Perimenis, 2005; Athanasopoulos et al., 2008; Athanasopoulos, 2010; Chapple, 2010; Füllhase et al., 2010). Moreover, there are no guidelines in antimuscarinic therapy in patients with a risk of urine retention. Overall, further study to investigate the benefit of antimuscarinic therapy in prostate hypertrophy is needed.

Risk of UTI between mirabegron and anti-muscarinic agents did not show a significant difference in our study, regardless of UTI history and sex. These results were consistent with a meta-analysis of clinical trials (Kelleher et al., 2018).

### Strengths and Limitations

To our knowledge, this study was the first to investigate the risk of UTI of OAB medications from a population-based source in Taiwan after mirabegron was covered by NHI. There are some strengths to our study. First, the data source NHIRD provides comprehensive medical records for almost the total population of Taiwan; thus, we were able to survey the whole picture rather than sampling studies. In addition, medical records decrease self-reporting and recall bias in community investigations. Second, the targeted population of the study was patients diagnosed with OAB, which represents that the disease was confirmed by a physician. Compared to a questionnaire-based study, our approach avoids some subjective interference. Third, we chose adherence as the indicator, which was the composite index between effectiveness and adverse effects and the key point for drug selection in guideline recommendations. Adherence, representing the balance of safety and effectiveness, indicates that the treatment is accepted by patients. Furthermore, we examined various indicators of adherence and performed subgroup and sensitivity analyses to confirm the robustness of the results.

Regardless, the study also has some limitations. First, we were unable to collect direct evaluation indicators, such as urinary diaries or changes in micturition, nocturia, and urgency. This prevented us from directly adjusting the disease severity among different populations. Despite our effort to reduce influence, such as by stratification or adjusting related variables in multiple regression, some confounding factors cannot be avoided within groups. The second limitation was that the NHIRD is a claims database; thus, prevalence may be underestimated, as some patients do not seek medical help. Last, according to information in the database, we cannot provide information on why the patients did not receive medication therapy.

Here, we provide the whole picture of OAB in Taiwan, and future studies on risk factors for UTI in patients who receive OAB treatment can be conducted.

## Conclusion

Our study showed there was no difference between anticholinergic medications and beta-3 agonists, nor between high and low adherence in the risk of UTI in recent years in Taiwan. The prevalence of OAB has increased recently. The risk of UTI for patients with high and low adherence and severe and mild UTI was not influenced by targeted drugs in our observed period.

## Data Availability

The datasets presented in this article are not readily available because data are available from the National Health Insurance Research Database (NHIRD) published by the Bureau of National Health Insurance (BNHI) of the Ministry of Health and Welfare. Requests to access the datasets should be directed to https://nhird.nhri.org.tw/.

## References

[B1] Abrams P. Cardozo L. Fall M. Griffiths D. Rosier P. Ulmsten U. (2002). The Standardisation of Terminology of Lower Urinary Tract Function: Report from the Standardisation Sub-committee of the International Continence Society. Neurourol Urodyn 21, 167–178. 10.1002/nau.10052 11857671

[B2] Andersson K. E. Arner A. (2004). Urinary Bladder Contraction and Relaxation: Physiology and Pathophysiology. Physiol. Rev. 84, 935–986. 10.1152/physrev.00038.2003 15269341

[B3] Athanasopoulos A. (2010). Antimuscarinics and Bladder Outlet Obstruction: from a Contraindication to an Indication? Neurourol Urodyn 29 (Suppl. 1), S46–S50. 10.1002/nau.20807 20127792

[B4] Athanasopoulos A. Giannitsas K. (2011). An Overview of the Clinical Use of Antimuscarinics in the Treatment of Overactive Bladder. Adv. Urol. 2011, 820816. 10.1155/2011/820816 21687579 PMC3114080

[B5] Athanasopoulos A. Mitropoulos D. Giannitsas K. Perimenis P. (2008). Safety of Anticholinergics in Patients with Benign Prostatic Hyperplasia. Expert Opin. Drug Saf. 7, 473–479. 10.1517/14740338.7.4.473 18613810

[B6] Athanasopoulos A. Perimenis P. (2005). Efficacy of the Combination of an Alpha1-Blocker with an Anticholinergic Agent in the Treatment of Lower Urinary Tract Symptoms Associated with Bladder Outlet Obstruction. Expert Opin. Pharmacother. 6, 2429–2433. 10.1517/14656566.6.14.2429 16259574

[B7] Chapple C. (2010). Antimuscarinics in Men with Lower Urinary Tract Symptoms Suggestive of Bladder Outlet Obstruction Due to Benign Prostatic Hyperplasia. Curr. Opin. Urol. 20, 43–48. 10.1097/MOU.0b013e3283330862 19875964

[B8] Chuang F. C. Kuo H. C. (2013). Increased Urothelial Cell Apoptosis and Chronic Inflammation Are Associated with Recurrent Urinary Tract Infection in Women. PLoS One 8, e63760. 10.1371/journal.pone.0063760 23691091 PMC3655152

[B9] Chuang Y. C. Liu S. P. Lee K. S. Liao L. Wang J. Yoo T. K. (2019). Prevalence of Overactive Bladder in China, Taiwan and South Korea: Results from a Cross-Sectional, Population-Based Study. Low Urin Tract Symptoms 11, 48–55. 10.1111/luts.12193 28967230 PMC7379992

[B10] Foxman B. (2014). Urinary Tract Infection Syndromes: Occurrence, Recurrence, Bacteriology, Risk Factors, and Disease burden. Infect. Dis. Clin. North. Am. 28, 1–13. 10.1016/j.idc.2013.09.003 24484571

[B11] Füllhase C. Soler R. Gratzke C. Brodsky M. Christ G. J. Andersson K. E. (2010). Urodynamic Evaluation of Fesoterodine Metabolite, Doxazosin and Their Combination in a Rat Model of Partial Urethral Obstruction. BJU Int. 106, 287–293. 10.1111/j.1464-410X.2009.09008.x 19888972

[B12] Gratzke C. van Maanen R. Chapple C. Abrams P. Herschorn S. Robinson D. (2018). Long-term Safety and Efficacy of Mirabegron and Solifenacin in Combination Compared with Monotherapy in Patients with Overactive Bladder: A Randomised, Multicentre Phase 3 Study (SYNERGY II). Eur. Urol. 74, 501–509. 10.1016/j.eururo.2018.05.005 29866467

[B13] Hsing A. W. Ioannidis J. P. (2015). Nationwide Population Science: Lessons from the Taiwan National Health Insurance Research Database. JAMA Intern. Med. 175, 1527–1529. 10.1001/jamainternmed.2015.3540 26192815

[B14] Karve S. Cleves M. A. Helm M. Hudson T. J. West D. S. Martin B. C. (2009). Good and Poor Adherence: Optimal Cut-point for Adherence Measures Using Administrative Claims Data. Curr. Med. Res. Opin. 25, 2303–2310. 10.1185/03007990903126833 19635045

[B15] Ke Q. S. Lee C. L. Kuo H. C. (2021). Recurrent Urinary Tract Infection in Women and Overactive Bladder - Is There a Relationship? Tzu Chi Med. J. 33, 13–21. 10.4103/tcmj.tcmj_38_20 33505873 PMC7821830

[B16] Kelleher C. Hakimi Z. Zur R. Siddiqui E. Maman K. Aballéa S. (2018). Efficacy and Tolerability of Mirabegron Compared with Antimuscarinic Monotherapy or Combination Therapies for Overactive Bladder: A Systematic Review and Network Meta-Analysis. Eur. Urol. 74, 324–333. 10.1016/j.eururo.2018.03.020 29699858

[B17] Kuo H. C. Liu H. T. Tyagi P. Chancellor M. B. (2010). Urinary Nerve Growth Factor Levels in Urinary Tract Diseases with or without Frequency Urgency Symptoms. Low Urin Tract Symptoms 2, 88–94. 10.1111/j.1757-5672.2010.00065.x 26676289

[B18] Lightner D. J. Gomelsky A. Souter L. Vasavada S. P. (2019). Diagnosis and Treatment of Overactive Bladder (Non-neurogenic) in Adults: AUA/SUFU Guideline Amendment 2019. J. Urol. 202, 558–563. 10.1097/JU.0000000000000309 31039103

[B19] Nik-Ahd F. Lenore Ackerman A. Anger J. (2018). Recurrent Urinary Tract Infections in Females and the Overlap with Overactive Bladder. Curr. Urol. Rep. 19, 94. 10.1007/s11934-018-0839-3 30215140

[B20] Sacco E. Bientinesi R. (2012). Mirabegron: a Review of Recent Data and its Prospects in the Management of Overactive Bladder. Ther. Adv. Urol. 4, 315–324. 10.1177/1756287212457114 23205058 PMC3491758

[B21] Szabo S. M. Gooch K. L. Walker D. R. Johnston K. M. Wagg A. S. (2018). The Association Between Overactive Bladder and Falls and Fractures: A Systematic Review. Adv. Ther. 35, 1831–1841. 10.1007/s12325-018-0796-8 30255417 PMC6223978

[B22] Takasu T. Ukai M. Sato S. Matsui T. Nagase I. Maruyama T. (2007). Effect of (R)-2-(2-aminothiazol-4-yl)-4'-{2-[(2-hydroxy-2-phenylethyl)amino]ethyl} Acetanilide (YM178), a novel Selective Beta3-Adrenoceptor Agonist, on Bladder Function. J. Pharmacol. Exp. Ther. 321, 642–647. 10.1124/jpet.106.115840 17293563

